# The Silent Abdomen: The Conservative Management of Large Idiopathic Pneumoperitoneum

**DOI:** 10.7759/cureus.109171

**Published:** 2026-05-19

**Authors:** Yifan Liu, Michael Auld, Geoffrey Stieler

**Affiliations:** 1 Medicine, University of Queensland, Brisbane, AUS; 2 Radiology, Ipswich Public Hospital, Ipswich, AUS; 3 Department of Surgery, Queen Elizabeth II Hospital, Brisbane, AUS

**Keywords:** computed tomography, idiopathic intestinal perforation, oral contrast, pnuemoperitoneum, visceral ischemia

## Abstract

Pneumoperitoneum is typically considered a sign of gastrointestinal perforation requiring emergency surgery. However, benign or idiopathic pneumoperitoneum can occur without peritonitis or systemic illness and may be managed without surgery. We report the case of an 82-year-old male with an incidentally discovered large-volume pneumoperitoneum (estimated at 2.2 litres) on imaging, despite the absence of abdominal pain, fever, or peritoneal signs. A CT scan with oral contrast demonstrated no evidence of perforation, and the patient remained clinically stable throughout admission. He was treated conservatively with antibiotics and bowel rest, achieving a full recovery without surgical intervention. This report highlights the importance of distinguishing benign or idiopathic pneumoperitoneum from true surgical emergencies, because in selected patients with reassuring clinical and radiological findings, conservative management may be a safe and effective alternative to unnecessary surgical exploration.

## Introduction

Pneumoperitoneum is defined as the presence of air within the abdominal cavity but outside the viscera. The diagnosis of pneumoperitoneum is often made using imaging, including plain radiographs or CT. Most patients with pneumoperitoneum present acutely with abdominal pain, often followed by sepsis due to gastrointestinal tract perforation, requiring emergency surgery to locate and repair the defect. However, patients with benign, non-surgical causes of pneumoperitoneum who show no signs of acute illness can often be managed conservatively [1]. This report describes the case of a patient with large-volume chronic pneumoperitoneum who was treated successfully without surgical intervention.

## Case presentation

An 82-year-old male was referred by his general practitioner to the emergency department due to an incidental pneumoperitoneum detected on CT. The index CT was obtained after concern for pneumoperitoneum arose from a lumbar plain film, which had been performed as part of an osteoporosis workup. The initial CT images are shown in Figure 1. The patient reported stable abdominal bloating over the past 12 months without other symptoms such as abdominal pain or fever. His medical history included hypertension, dyslipidaemia, and chronic bladder outlet obstruction, but no prior abdominal surgeries. On examination, the patient’s abdomen was grossly distended with hyperresonance on percussion, but there were no signs of tenderness or peritonism. Blood work revealed a normal white cell count (WCC) of 7.7 x 10⁹/L (reference range: 3.5-11 x 10⁹/L) and a mildly elevated C-reactive protein (CRP) of 5.3 mg/L (reference: <5.0 mg/L).

**Figure 1:**
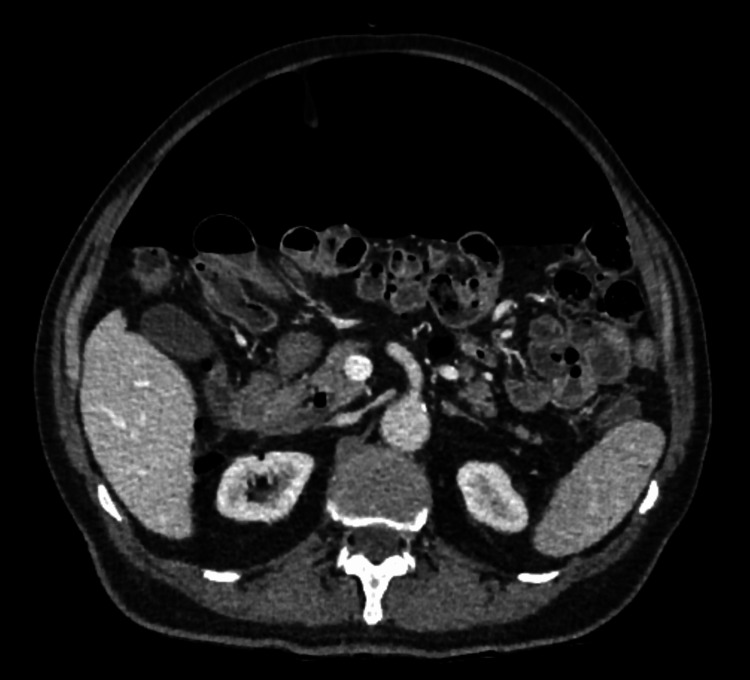
Axial slice of CT abdomen with portal venous contrast showing unexpected, large-volume pneumoperitoneum CT: computer tomography

An abdominal plain film (Figure 2) and a second CT with oral contrast (Figures 3, 4) were performed and revealed no evidence of contrast extravasation. The volume of pneumoperitoneum was estimated at 2.2 litres using the Siemens Syngo.via freehand contouring software tool (Siemens Healthineers, Erlangen, Germany). This process involved manually outlining the relevant anatomical contours and interpolating the selected slices to generate a reasonably accurate volume measurement. Given the benign clinical presentation, benign pneumoperitoneum was considered the most likely diagnosis, with perforated viscus deemed unlikely. The patient was managed nonoperatively with intravenous ceftriaxone and metronidazole, along with bowel rest. Owing to his clinical stability, he was discharged on the second day of admission. At the six-week follow-up, he remained well and asymptomatic.

**Figure 2:**
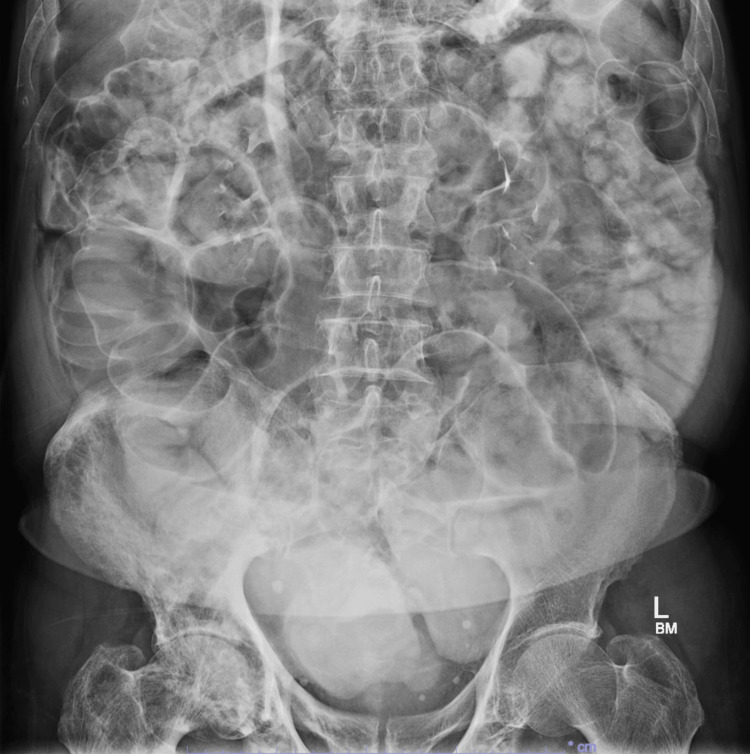
Supine abdominal X-ray showing Rigler sign (also known as double-wall sign suggestive of pneumoperitoneum)

**Figure 3:**
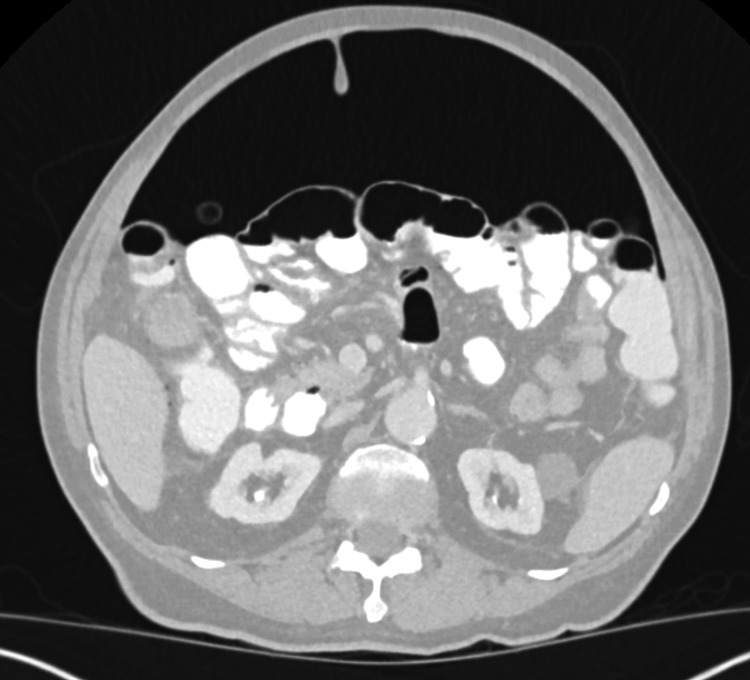
Axial slice of CT abdomen with oral contrast showing no contrast extravasation to localise gastrointestinal perforation CT: computer tomography

**Figure 4:**
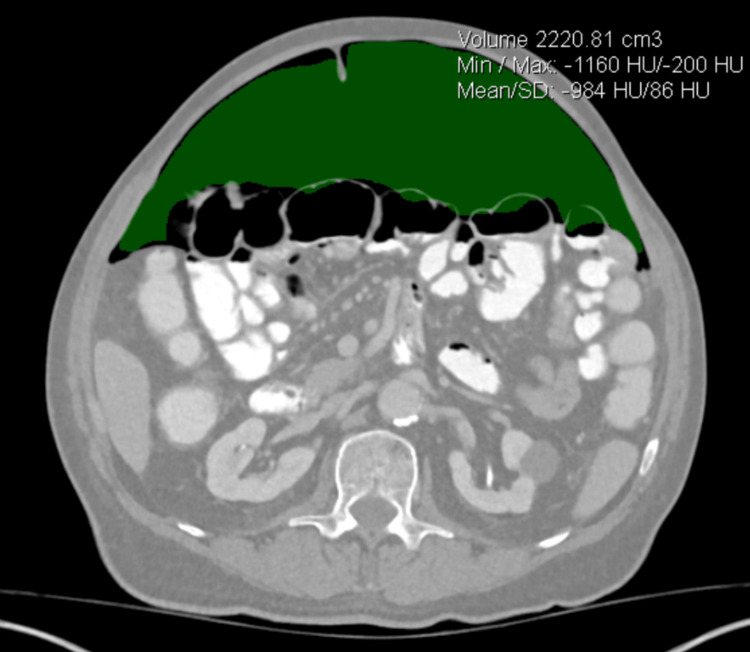
Total pneumoperitoneum volume estimated at ~2200 mL using Siemens Syngo.via freehand contouring software

## Discussion

Benign pneumoperitoneum is exceedingly rare, with the most common cause being pneumatosis cystoides intestinalis (PCI) [1]. PCI is characterised by the presence of submucosal and/or subserosal cysts containing gas, often ranging from 5 mm to 20 mm in size. It is hypothesised that the cysts arise either from excess hydrogen gas produced by intraluminal bacteria (the bacterial theory) or from gas translocation due to increased luminal pressure (the mechanical theory) [2]. Hakim et al. reported a case of benign pneumoperitoneum in a patient with small intestinal bacterial overgrowth (SIBO), who was successfully managed with oral metronidazole and a low-carbohydrate diet [3].

While most cases of PCI are benign, concern for perforated viscus or necrotising enterocolitis often results in unnecessary surgical exploration. Rupture of these cysts does not constitute a true perforation, as they are confined to the submucosal or subserosal layers and do not communicate with the intestinal lumen. The literature has also reported associations between PCI and connective tissue diseases such as systemic sclerosis, as well as chemotherapy and hormonal therapy [4]. Complications of PCI are rare but can include intestinal volvulus, obstruction, and haemorrhage [5]. It is crucial to distinguish PCI from pneumatosis intestinalis, which may result from bowel ischemia or infarction, bowel obstruction with increased intraluminal pressure, or severe bowel inflammation (including infectious colitis, inflammatory bowel disease, or diverticulitis).

Other benign causes of pneumoperitoneum can be classified as thoracic, female genital tract, iatrogenic, or idiopathic. Air from the mediastinum can directly track into the retroperitoneal space and peritoneal cavity. Thoracic causes of benign pneumoperitoneum include trauma such as barotrauma, pneumothorax, cardiopulmonary resuscitation, and invasive ventilation with high peak inspiratory pressures [6]. Kubina et al. reported a case of benign pneumoperitoneum resulting from an alveolar-peritoneal fistula with subperitoneal emphysema tracking along the diaphragm over the liver [7]. A diaphragmatic defect likely allowed intrathoracic air to escape through pleural and diaphragmatic openings.

The female genital tract can also permit atmospheric air to enter the peritoneal cavity. An anatomical communication exists between the endometrium and the peritoneum via the fallopian tubes. This communication is often demonstrated during infertility evaluations using a hysterosalpingogram. The procedure is typically performed during the proliferative phase of the menstrual cycle, when the endometrium is thinnest, to improve visualisation [8]. Water-soluble iodinated contrast is injected into the uterus to assess tubal patency by confirming intraperitoneal spillage. Because of this anatomical pathway, cases of benign pneumoperitoneum following sexual intercourse and vaginal douching have been reported [9].

Iatrogenic causes of benign pneumoperitoneum are often related to a patient’s post-laparoscopy or post-laparotomy status. Insufflation of the abdomen with carbon dioxide during laparoscopic surgery is usually minimised at the end of the procedure by applying external abdominal pressure. However, this approach is not always completely effective, and residual intraperitoneal gas may be detected on postoperative imaging. It is therefore important to clearly document recent surgery on the imaging request to avoid misinterpreting pneumoperitoneum as secondary to a perforated viscus. Maheshwari et al. reported a case of benign pneumoperitoneum in a peritoneal dialysis patient with abdominal pain [10]. In this case, air was removed by placing the patient in the Trendelenburg position and attaching a peritoneal dialysis drain bag. Other iatrogenic causes include diagnostic and therapeutic colonoscopy, including procedures involving argon plasma coagulation.

True idiopathic benign pneumoperitoneum is exceedingly rare, with only a few documented cases in the literature. These patients often present with vague abdominal symptoms, including distention, with or without abdominal pain, but without signs of sepsis or peritonitis. The diagnosis is often incidental, identified on imaging studies performed for unrelated reasons. Pouli et al. estimated that CT, with or without oral contrast, can predict the location of a potential perforation with an accuracy of 86% [11]. Sites of perforation are typically accompanied by CT findings such as inhomogeneous mural enhancement, wall thickening, fat stranding, mural defects, and leakage of oral contrast.

After the presence of pneumoperitoneum is established, surgeons are almost always consulted, and benign causes should be considered to avoid unnecessary emergency laparoscopy or laparotomy. A detailed history and physical examination, combined with CT findings, often provide important clues regarding the nature of the pneumoperitoneum. Management of benign pneumoperitoneum typically includes bowel rest, with or without total parenteral nutrition, intravenous antibiotics, and interval imaging. Diagnosing benign pneumoperitoneum can be challenging if patients present with sepsis unrelated to the abdomen. Any signs of clinical deterioration may necessitate surgical intervention to exclude acute viscus perforation.

## Conclusions

Idiopathic pneumoperitoneum is a rare condition that can present a diagnostic challenge. Acute perforation of abdominal viscera is often associated with characteristic CT findings, which can help surgeons identify the site of perforation. We presented the case of a patient with large-volume idiopathic pneumoperitoneum without any evidence of acute perforation on CT, who was successfully managed conservatively. This benign pathology should always be considered in the differential diagnosis, particularly in asymptomatic and clinically stable patients, to avoid unnecessary surgical intervention.
